# Supplementary material for: Complexome profiling of the Chlamydomonas psb28 mutant reveals TEF5 as an early PSII assembly factor
Source: Plant Cell. 2025 Mar 17;37(6):koaf055. doi: 10.1093/plcell/koaf055 (PMC12164590; doi:10.1093/plcell/koaf055)
Supplement: koaf055_Supplementary_Data [file koaf055_supplementary_data.zip › psb28_interactive.zip!/plots/intensity/qualityPlots/peptides/_Cre17.g731571.t1.1.html]

### Cre17.g731571.t1.1

Trivial name:   
SeqCoverage [median]: 13.00  
Mass: 16.28 kDa  
  
 

| Sequence | MissedCleavage | Mass [Da] | wtIntensity | muIntensity | Score |
| --- | --- | --- | --- | --- | --- |
| APQPQAAEAQEAAAQEEAKR | 1 | 2093.01 | 0.00 | 644.87 | 102.37 |
|  |  |  | 0.0 | 644.9 |  |
